# The Association Between Glymphatic System Dysfunction and Cognitive Impairment in Cerebral Small Vessel Disease

**DOI:** 10.3389/fnagi.2022.916633

**Published:** 2022-06-24

**Authors:** Jie Tang, Miaoyi Zhang, Na Liu, Yang Xue, Xue Ren, Qi Huang, Langfeng Shi, Jianhui Fu

**Affiliations:** Department of Neurology, Huashan Hospital, Fudan University, Shanghai, China

**Keywords:** cerebral small vessel disease, vascular cognitive impairment (VCI), glymphatic system, diffusion tensor image (DTI), white matter lesions (WMLs)

## Abstract

The mechanism of cognitive impairment in patients with cerebral small vessel disease (CSVD) remains unknown. The glymphatic system dysfunction, which has been demonstrated to influence cognitive impairment, can be evaluated by diffusion tensor image analysis along the perivascular space (ALPS index). We explored whether cognitive impairment in CSVD is associated with glymphatic clearance dysfunction. In this study, 133 patients with CSVD were enrolled and underwent neuropsychological test batteries as well as magnetic resonance imaging (MRI). They were then categorized into a CSVD with cognitive impairment (CSVD-CI) group and a cognitively normal CSVD (CSVD-CN) group. The ALPS index and four CSVD markers [white matter lesions (WMLs), cerebral microbleeds (CMBs), lacunes, and perivascular spaces (PVSs)] were also assessed. Univariate analysis showed that the ALPS index was significantly different between the CSVD-CN (*n* = 50) and CSVD-CI groups (*n* = 83) (*p* < 0.001). This difference remained significant (95% *CI* < 0.001–0.133) after adjusting for six common risk factors (age, education, hypertension, diabetes, smoking, and alcohol abuse) as well as CSVD markers. The ALPS index was independently linearly correlated with global cognitive function, executive function, attention function, and memory after adjusting for the aforementioned six risk factors or CSVD markers. Our results suggest that glymphatic system impairment is independently related to cognitive impairment in patients with CSVD.

## Introduction

Cerebral small vessel disease (CSVD) is common among older people. Cognitive impairment is one of the most important manifestations of CSVD. Vascular cognitive impairment and vascular dementia constitute the second most common cause of cognitive impairment. However, the factors causing cognitive impairment remain unknown. White matter lesions (WMLs) and lacunes, which are classical CSVD markers, are related to cognitive impairment in CSVD and could be used to predict cognitive impairment (Hamilton et al., 2021). However, in the clinic, some CSVD patients with mild WMLs have a severe cognitive impairment, which suggests that there are still some important factors that contribute to cognitive impairment in CSVD aside from traditional imaging markers and other imaging markers might enable predictions of cognitive impairment caused by CSVD.

Commonly, cognitive impairment in patients with CSVD occurs due to cerebral hypoperfusion or because other blood components permeate into the brain through a broken blood–brain barrier (BBB). Both of these situations are harmful to neurons and neuroglia (Wardlaw et al., 2013a). In addition, many studies have found that amyloid-beta (Aβ) protein is deposited in the brains of patients with CSVD (Ye et al., 2015). In addition, the deposition of toxins leads to neuronal dysfunction or death in the brain and contributes to cognitive decline. In addition to Aβ, other toxins might also accelerate cognitive impairment.

Recently, the glymphatic system was discovered. With the help of Aquaporin 4, cerebrospinal fluid (CSF) flow in the periarterial space enters the brain and becomes interstitial fluid (ISF). It was then drained orderly through the perivenous space, meningeal or olfactory mucosal lymphatics, cervical lymphatic vessel, and finally returned to the peripheral venous. This CSF-ISF exchange system is called the glymphatic system, and its main role includes metabolic product transportation and metabolic waste elimination (Nedergaard and Goldman, 2020). In patients with Alzheimer's disease (AD) and animal models, dysfunction of the glymphatic system contributes to the deposition of Aβ and cognitive impairment (Reeves et al., 2020). CSVD leads to arteriosclerosis and vascular pulsation (Fu et al., 2006), which are the driving forces of the glymphatic system (Nedergaard and Goldman, 2020). In addition, some patients with CSVD have comorbid sleep disorders, and changes in sleep rhythm also impair the function of the glymphatic system (Hablitz et al., 2020; Chong et al., 2022). Our animal study verified the dysfunction of the glymphatic system in a rat model of CSVD (Xue et al., 2020). These studies indicated that damage to the glymphatic system might be another factor that leads to cognitive impairment in patients with CSVD.

It is difficult to evaluate the glymphatic system in the human body directly. In 2017, based on diffusion tensor imaging (DTI), Taoka et al. proposed the DTI analysis along the perivascular space (ALPS) index to evaluate the glymphatic system function. The ALPS index was calculated by the diffusivity at a site beside the lateral ventricle body at which projection and association fibers are orthogonal to the perivascular space (PVS; Taoka et al., 2017). More recently, Lou et al. compared this method with classical enhanced glymphatic magnetic resonance imaging (MRI) by the intrathecal administration of gadolinium, and they confirmed that the ALPS index could reflect glymphatic clearance function (Zhang et al., 2021).

To further explore the reason underlying cognitive impairment in patients with CSVD and identify other new imaging markers of cognitive impairment in patients with CSVD, we studied the relationship of the ALPS index and cognitive impairment in these patients.

## Materials and Methods

### Patients

Patients who visited the clinic at the Neurology Department of Huashan Hospital and met the inclusion and exclusion criteria below were prospectively consecutively enrolled in our study from Mar 2015 to Mar 2019.

Subjects were enrolled if they met the following criteria: (1) Fazekas 2–3 WMLs and at least one of the following three markers: lacune, cerebral microbleed (CMB), and PVS markers; (2) aged 50–80 years old. The exclusion criteria were as follows: (1) cerebral infarction with a diameter of infarction core larger than 2 cm; (2) cortical infarction; (3) stroke due to any potential cardioembolic source; (4) lacunar stroke syndrome within 6 months; (5) cerebrovascular stenosis with >50% luminal stenosis suggested by transcranial Doppler ultrasound, computed tomography angiography or magnetic resonance angiography; (6) genetically confirmed hereditary CSVD or suspected hereditary CSVD according to the family history and clinical features; (7) potential inflammatory and immunologically mediated CSVD according to clinical features and immune index; (8) WMLs that might be caused by other reasons except CSVD, such as multiple sclerosis; (9) cognitive impairment that might be caused by other diseases, such as neurodegeneration disease or hydrocephalus; (10) severe depression or other affective disorder that affects cognitive evaluation; (11) language disturbances that impair communication; and (12) MRI contraindications.

All demographic information, vascular risk information, and history of diseases or medicine were recorded. This study was approved by the Institutional Review Board of Huashan Hospital. All participants signed informed consent documents.

### Cognitive Function Evaluation

All subjects underwent a neuropsychological test battery. All evaluations were administered by an experienced clinical psychologist who was blinded to the clinical and imaging data. The Montreal Cognitive Assessment (MoCA) was used for global cognitive function evaluation. Memory function was assessed by the total number of five recalls in the Auditory Verbal Learning Test (AVLT-sum), including three immediate recalls, one short delayed recall and one long delayed recall. Executive function was evaluated by the difference time value between the Trail Making Test B and A time [TMT (B-A)] and the total Verbal Fluency Test (VFT) score. Our VFT included animal, fruit, and vegetable categories, in which as many names as possible must be mentioned within 60 s. Attention function was assessed by the Symbol Digit Modalities Test (SDMT), language function was evaluated by the Boston Naming Test (BNT), and visuospatial function was assessed by the copy trial of Rey's Complex Figure Test (RCFT-C). The cutoff MoCA scores to identify vascular cognitive impairment were 13/14, 19/20, and 24/25 for those who had received 0, 1–6, and 7 or more years of education, respectively (Lu et al., 2011). Based on the MoCA score, the cognitive diagnosis of the patients with CSVD was finally determined by the consensus meetings, which were attended by a neurologist, a clinical psychologist, a neuroradiologist, and a nurse.

### MRI

All patients with CSVD in our study were scanned using a 3-T MRI scanner (Siemens Magneton Verio 3T). A dedicated 8-channel head coil was equipped for radiofrequency signal transmission and reception. The imaging protocol included T1-weighted and T2-weighted imaging, fluid-attenuated inversion recovery (FLAIR) imaging, three-dimensional brain volume (3D-BRAVO) analysis, and susceptibility-weighted imaging (SWI), DTI. The 3D-BRAVO parameters were as follows: repetition time (TR) = 8.8 ms, echo time (TE) = 1.0 ms, flip angle = 15 degrees, slice thickness = 1 mm, field of view (FOV) = 320 mm, matrix = 320 × 320, and voxel size = 1 × 1 mm. The T2-weighted imaging parameters were TR = 9,675 ms, TE = 150 ms, slice thickness = 2 mm, FOV = 240 mm, and matrix = 448 × 320. The T2-FLAIR parameters were TR = 4,480 ms, TE = 120 ms, slice thickness = 5 mm, FOV = 230 mm, and matrix = 480 × 480. The DTI parameters were TR = 17,000 ms, TE = 86.2 ms, FOV = 224 mm, matrix = 128 × 128, slice thickness = 2.4 mm, 20 directions and b = 1,000 s/mm^2^. The SWI parameters were TR = 62.1 ms, TE = 32 ms, flip angle = 15°, slice thickness = 1.6 mm, FOV = 240 mm, and matrix = 240 × 240.

### Traditional CSVD Marker Evaluation

Cerebral small vessel disease markers were rated by two experienced neurologists who were blinded to the clinical data according to the Standards for Reporting Vascular Changes on Neuroimaging (STRIVE) criteria (Wardlaw et al., 2013b).

White matter lesions were defined as regions that were hyperintense on T2-weighted and T2-FLAIR imaging and were not hypointense on T1-weighted imaging. WMLs were rated visually on axial T2-FLAIR images using the modified Fazekas scale. The severity of WMLs in both the centrum semiovale and periventricular regions was rated as level 1 (mild), level 2 (moderate), and level 3 (severe; Pantoni et al., 2005). Then, the severity ratings of the two areas were summed.

Cerebral microbleeds (CMBs) were defined as hyperintense, round lesions that were smaller than 10 mm on SWI scans. CMBs were counted in the lobar, deep, and infratentorial regions, and then the total CMBs were recorded (Gregoire et al., 2009).

Lacunes were defined as round or approximately round lesions, 3–15 mm in diameter, that were hyperintense on T2-weighted imaging, hypointense on T1-weighted imaging, and hypointense with a rim of hyperintensity on T2-FLAIR imaging without hyperintensity on SWI scans. The lacunes were counted in the lobar, deep and infratentorial regions. Then, the total number of lacunes was recorded (Pasi et al., 2017).

Perivascular spaces were defined as linear, round, or ovoid regions of signal with an intensity similar to that of CSF on all sequences and a diameter usually smaller than 3 mm. To distinguish PVSs from small lacunae, PVSs were defined as not having a hyperintense rim on T2-FLAIR images. The severity of PVSs in the centrum semiovale and basal ganglia was rated according to the number of spaces in a slice from the side containing the larger quantity of PVSs: level 0, none; level 1, 1–10; level 2, 11–20; level 3, 21–40; and level 4, > 40 (Potter et al., 2015). Either centrum semiovale or basal ganglia severity >2 was regarded as moderate to severe PVS (Banerjee et al., 2017).

### ALPS Index Calculation

The free FMRIB software library (FSL) diffusion toolbox (FDT) was used to calculate the diffusion metric images. The ALPS index was calculated as described in a previous report (Taoka et al., 2017). First, a diffusion tensor at each voxel of the brain was estimated and outputted as a series of images, such as color-coded fractional anisotropy (FA), mean diffusivity (MD), and diffusivity maps with *x*-, *y*-, and *z*-axes (Dxx, Dyy, and Dzz). These diffusion tensors were used to measure the diffusivity along the PVS direction by means of comparison with those of projection fibers and association fibers in a slice through the body of the lateral ventricle. At this level, the PVS direction was perpendicular to the ventricle wall. As a result, the PVS direction at this level was parallel to the *x*-axis (right–left), mostly in the axial plane. This direction is also perpendicular to the directions of both the association fibers (mostly on the *y*-axis) and the projection fibers (mostly on the *z*-axis). Thus, the diffusivity along the *x*-axis at areas with projection/association fibers at least partially represents the diffusivity along the PVSs. Then, a 5 mm diameter spherical region of interest (ROI) within the area of the projection fibers, the association fibers, and the subcortical fibers in the left hemisphere was placed on a color-coded FA map of the plane at the level of the lateral ventricle body. Furthermore, the ALPS index that reflected the activity of the glymphatic system in individual cases was calculated. That is, the ratios of the mean *x*-axis diffusivity in the area of the projection fiber (Dxproj) and *x*-axis diffusivity in the area of association fibers (Dxassoc) to the mean *y*-axis diffusivity in the area of the projection fiber (Dyproj) and *z*-axis diffusivity in the area of association fibers (Dzassoc) were determined. In brief, ALPS index = mean (Dxproj, Dxassoc)/mean (Dyproj, Dzassoc).

In the area of the projection fibers, the dominant fibers run along the direction of the *z*-axis, and the *x*- and *y*-axes are perpendicular to the dominant fibers. Similarly, in the area of the association fibers, the dominant fibers run along the direction of the *y*-axis, and both the *x*- and *z*-axes are perpendicular to the dominant fibers. The major difference in the behavior of water molecules between the *x*-axis diffusivity in these areas (Dxproj and Dxassoc) and the diffusivity perpendicular to them (Dyproj and Dzassoc) was the existence of PVSs. Therefore, the ALPS index was calculated by measuring the diffusivity from the composite along the direction of the PVSs in perpendicular association fibers and projection fibers to reflect the function of the glymphatic system.

### Statistics

For the statistical analysis, SPSS 18.0 (IBM Inc., Chicago, IL, USA) was used. For categorical variables, the differences between two groups were assessed by the chi-square test or continuity correction test. For continuous variables, the differences between two groups were compared using the Mann–Whitney *U*-test. Backward stepwise multivariable logistic regression models were used to evaluate variables that were independently associated with cognitive impairment. The area under the receiver operating characteristic curve (AUROC) was used to evaluate the ability of the model. Multivariate regression linear analysis was further performed to determine the factors that were linearly correlated with each neuropsychological test.

## Results

A total of 133 patients with CSVD were enrolled in our study. Among the 133 patients with CSVD, 83 had cognitive impairment (CSVD-CI), and 50 were cognitively normal (CSVD-CN). The demographic data are shown in Table 1. The patients with CSVD-CN and CSVD-CI averaged 64.45 ± 7.768 and 66.18 ± 7.575 years of age, respectively, and the length of their education averaged 10.21 ± 4.247 and 8.78 ± 4.749 years, respectively. Additionally, 64.0 and 63.9% of the participants in the CSVD-CN group and the CSVD-CI group, respectively, were male. The prevalence of hypertension in patients in the CSVD-CN and CSVD-CI groups was 87.8 and 92.9%, respectively, and the percentages of patients in the CSVD-CN and CSVD-CI groups with a history of diabetes mellitus were 17.15 and 25.0%, respectively. The smoking rate of patients in the CSVD-CN group was 15.0%, and that of patients in the CSVD-CI group was 23.2%. The alcohol abuse rate was 10% in the CSVD-CN group and 14.0% in the CSVD-CI group. There was no difference between the patients in the CSVD-CN and CSVD-CI groups in age, education, sex, hypertension, diabetes mellitus, smoking rate, or alcohol abuse rate.

**Table 1:** Demographics, imaging and cognitive condition of participant.

	**CN (*n* = 50)**	**CI (*n* = 83)**	***p***
Age, year (mean ± SD)	64.45 ± 7.768	66.18 ± 7.575	0.224
Male, *n* (%)	32 (64.0%)	53 (63.9%)	1.000
Education year (mean ± SD)	10.21 ± 4.247	8.78 ± 4.749	0.060
Hypertension, *n* (%)	36 (87.8%)	52 (92.9%)	0.487
Diabetes, *n* (%)	7 (17.15%)	14 (25.0%)	0.456
Smoking, *n* (%)	6 (15.0%)	13 (23.2%)	0.437
Alcohol abuse, *n* (%)	4 (10.0%)	8 (14.0%)	0.756
WMLs, *n* (%)			<0.001
3	9 (18.0%)	7 (8.5%)	
4	16 (32.0%)	11 (13.4%)	
5	14 (28.0%)	11 (13.4%)	
6	11 (22.0%)	53 (64.6%)	
Number of lacunes (mean ± SD)	2.36 ± 2.593	3.27 ± 4.067	0.496
Number of CMBs (mean ± SD)	8.80 ± 13.178	20.14 ± 26.623	0.003
Moderate to severe PVS (mean ± SD)	38 (88.4%)	69 (86.3%)	0.788
ALPS-index (mean ± SD)	1.054 ± 0.142	0.958 ± 0.088	<0.001
MoCA (mean ± SD)	24.41 ± 4.036	16.34 ± 5.867	<0.001
TMT(B-A) (mean ± SD)	151.04 ± 121.550	206.76 ± 136.990	0.010
AVLT-sum (mean ± SD)	21.43 ± 11.793	10.7 ± 8.581	<0.001
SDMT (mean ± SD)	31.50 ± 11.630	16.82 ± 10.741	<0.001
VFT (mean ± SD)	41.2 ± 13.034	31.79 ± 14.758	0.001
BNT (mean ± SD)	20.54 ± 4.135	18.28 ± 4.979	0.017
RCFT-C (mean ± SD)	30.70 ± 4.903	23.53 ± 9.744	<0.001

*ALPS-index, diffusion tensor image analysis along the perivascular space; WMLs, White Matter Lesions; CMBs, Cerebral Microbleeds; PVS, perivascular space; MoCA, Montreal Cognitive Assessment; AVLT-sum, total number of five times recall of three trial Auditory Verbal Learning Test; TMT (B-A), time of Trail Making Test B- time of Trail Making Test A; VFT, Category Verbal Fluency Test; SDMT, Symbol Digit Modalities Test; RCFT-C, Rey's Complex Figure Test copy trail; BNT, Boston Naming Test*.

**Figure 1:**
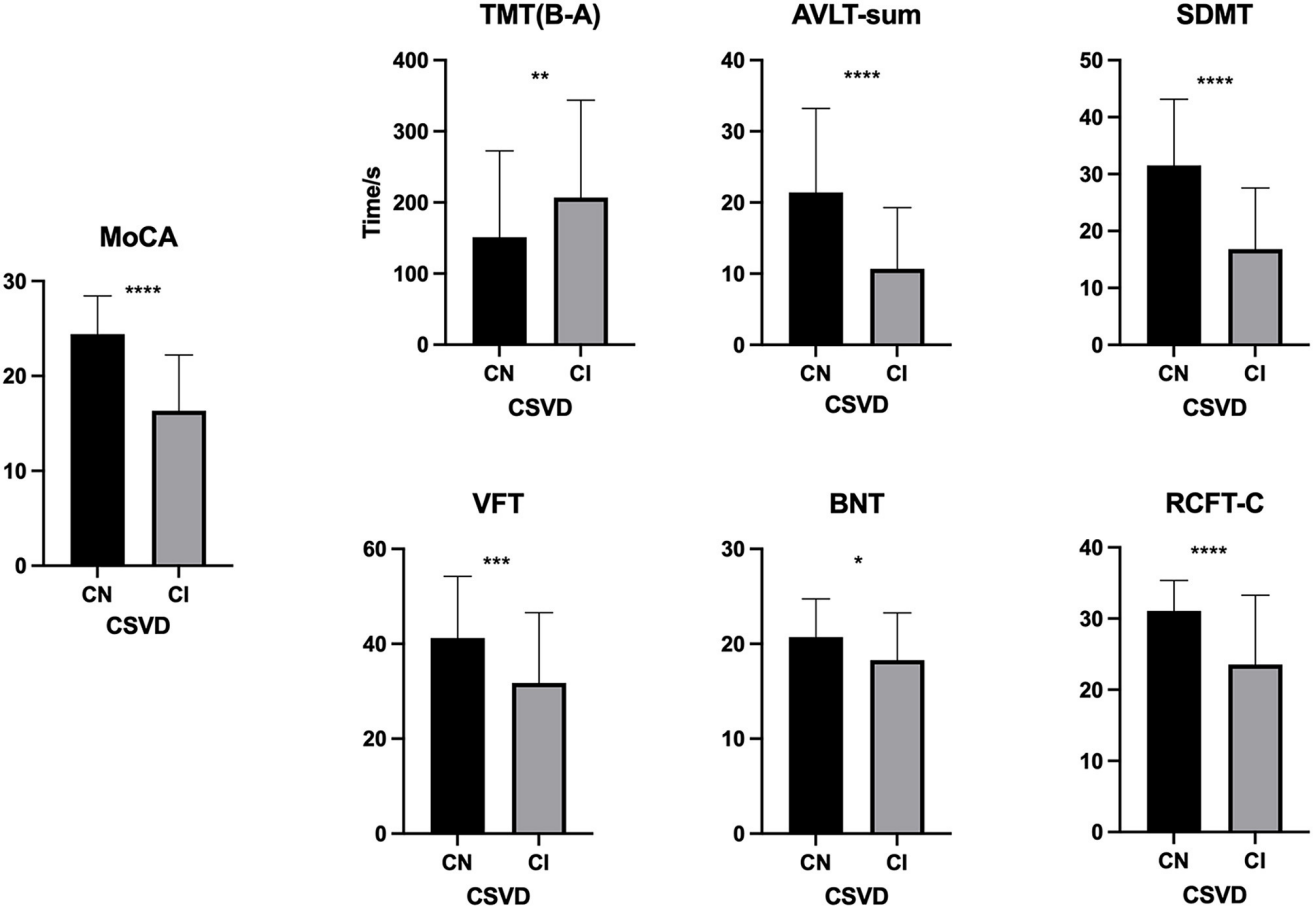
Comparison of neuropsychological test results between the cognitive normal (CN) and cognitive impairment (CI) groups. The difference between the CN and CI groups in the Montreal Cognitive Assessment (MoCA), the Trail Making Test B and A time [TMT (B-A)], the Auditory Verbal Learning Test (AVLT)-sum, the Symbol Digit Modalities Test (SDMT), the Verbal Fluency Test (VFT), the Boston Naming Test (BNT), and the Rey's Complex Figure Test (RCFT-C). *p < 0.05, **p ≤ 0.01, ***p ≤ 0.001, and ****p ≤ 0.0001.

As shown in Table 1, the mean (±SD) ALPS index in the CSVD-CN and CSVD-CI groups was 1.054 ± 0.142 and 0.958 ± 0.088, respectively; these values were significantly different (*p* < 0.001). For the traditional CSVD imaging marker, in univariate analysis, there were significant differences between patients with CSVD-CN and CSVD-CI in WML severity (*p* < 0.001) and the number of CMBs (*p* = 0.003). However, there were no differences in lacunes (*p* = 0.496) or PVSs (*p* = 0.788). All neuropsychological test results, such as MoCA (*p* < 0.001), TMT (B-A; *p* = 0.010), AVLT-sum (*p* < 0.001), SDMT (*p* < 0.001), VFT (*p* = 0.001), BNT (*p* = 0.017), and RCFT-C (*p* < 0.001), showed remarkable differences between patients in the CSVD-CN and CSVD-CI groups (Table 1 and Figure 1).

**Figure 2:**
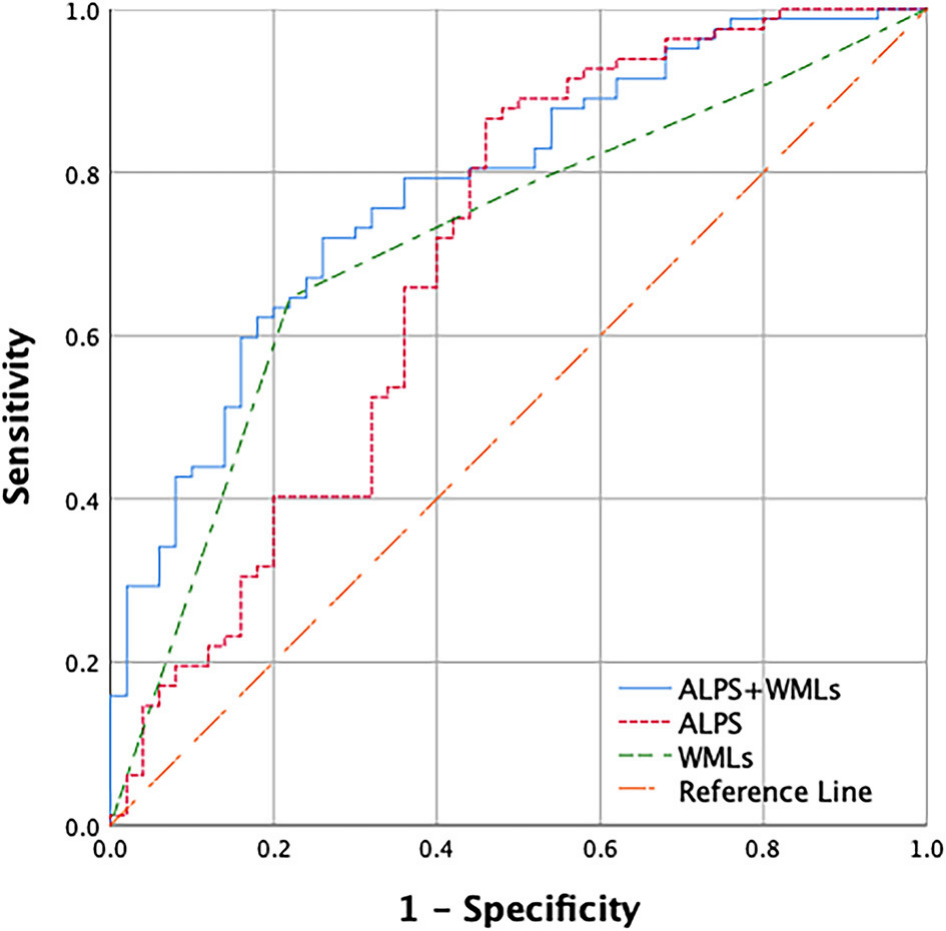
Receiver operating characteristic (ROC) curves used to predict cerebral small vessel disease (CSVD) cognitive impairment with the combination of the diffusion tensor image analysis along the perivascular space (ALPS) index and WMLs. Only the ALPS index and white matter lesions (WMLs) remained in the backward stepwise logistic regression model. The area under the receiver operating characteristic (AUROC) of the ALPS index and WMLs combined is 0.780 (blue), the AUROC of WMLs alone is 0.714 (green), and the AUROC of the ALPS index alone is 0.694 (red).

As shown in Table 2, the backward stepwise multivariable logistic regression model for cognitive impairment eliminated age, education, hypertension, diabetes, smoking, alcohol abuse, the number of lacunes, the number of CMB, and moderate to severe PVS; in the end, only the ALPS index and WMLs remained in the model. In this model, both the ALPS index (*p* = 0.005) and WMLs (*p* = 0.003) were significantly different between the patients in the CSVD-CN and CSVD-CI groups. Furthermore, as shown in Figure 2, we calculated the AUROC of this model combining the ALPS index and WMLs. The AUROC of the model, at 0.780, was greater than the AUROC of the ALPS index alone (0.694) or WMLs alone (0.714).

**Table 2:** Backward stepwise logistic regression analyses for CSVD cognitive impairment.

	**B**	**OR (95% C.I.)**	***P***
ALPS-index	−6.534	0.001 (<0.001–0.133)	0.005
WMLs	0.734	2.082 (1.294–3.354)	0.003

*Adjusted for age, education, hypertension, diabetes, smoking, alcohol abusing, the number of lacunes, the number of CMBs, and the severity of PVS. 95% CI, 95% confidence interval; ALPS-index, diffusion tensor image analysis along the perivascular space; WMLs, white matter lesions*.

To further explore the relationship between the ALPS index and cognitive impairment in patients with CSVD, using multivariate regression linear analysis our results showed that after adjusting for vascular risk factors of age, education, hypertension, diabetes mellitus, smoking, and alcohol abuse, the ALPS index was positively related to the MoCA (*p* = 0.005), score in the AVLT-sum (*p* = 0.002), the right number of SDMT (*p* < 0.001), and the VFT score (*p* = 0.008), and was negatively related to TMT (B-A; *p* = 0.010) (Table 3). However, there were no significant linear associations among the BNT (*p* = 0.814), RCFT-C (*p* = 0.526), and ALPS index (Table 3).

**Table 3:** Multivariate regression linear analysis between neuropsychological test in CSVD and vascular risks.

	**B (95%CI)**	***P***	**VIF**
MoCA			
ALPS-index	16.094 (5.064 to 27.123)	0.005	1.290
Age	0.094 (−0.089 to 0.277)	0.310	1.156
Education	0.683 (0.398 to 0.968)	<0.001	1.042
Hypertension	−1.808 (−5.505 to 1.889)	0.334	1.211
Diabetes	−0.292 (−3.483 to 2.899)	0.856	1.100
Smoking	0.793 (−3.186 to 4.771)	0.693	1.593
Alcohol abuse	−0.254 (−5.250 to 4.742)	0.920	1.650
TMT(B-A)			
ALPS-index	−252.082 (−441.516 to −62.647)	0.010	1.266
Age	1.921 (−1.629 to 5.472)	0.285	1.186
Education	−7.156 (−13.146 to −1.167)	0.020	1.066
Hypertension	62.526 (1.247 to 123.805)	0.046	1.208
Diabetes	0.268 (−61.498 to 62.035)	0.993	1.101
Smoking	−26.107 (−99.679 to 47.465)	0.483	1.498
Alcohol abuse	137.343 (42.367 to 232.318)	0.005	1.524
AVLT-sum			
ALPS-index	25.914 (9.687 to 42.140)	0.002	1.283
Age	0.160 (−0.135 to 0.455)	0.284	1.174
Education	0.603 (0.104 to 1.101)	0.018	1.079
Hypertension	−5.964 (−11.233 to −0.696)	0.027	1.201
Diabetes	0.867 (−4.204 to 5.938)	0.735	1.113
Smoking	−3.089 (−9.421 to 3.243)	0.335	1.551
Alcohol abuse	−2.662 (−10.777 to 5.453)	0.517	1.609
SDMT			
ALPS-index	43.740 (21.540 to 65.939)	<0.001	1.372
Age	−0.091 (−0.459 to 0.278)	0.627	1.216
Education	1.203 (0.586 to 1.820)	<0.001	1.057
Hypertension	−8.334 (−16.278 to −0.390)	0.040	1.238
Diabetes	−2.977 (−9.205 to 3.250)	0.344	1.103
Smoking	0.609 (−6.959 to 8.176)	0.873	1.456
Alcohol abuse	−4.824 (−14.795 to 5.147)	0.339	1.558
VFT			
ALPS-index	27.108 (7.411 to 46.804)	0.008	1.359
Age	0.006 (−0.319 to 0.331)	0.971	1.201
Education	0.672 (0.132 to 1.212)	0.015	1.098
Hypertension	−9.226 (−15.919 to −2.532)	0.008	1.166
Diabetes	−2.916 (−8.175 to 2.343)	0.273	1.109
Smoking	−1.928 (−8.280 to 4.424)	0.547	1.508
Alcohol abuse	−5.996 (−14.165 to 2.173)	0.148	1.614
BNT			
ALPS-index	0.975 (−7.223 to 9.173)	0.814	1.344
Age	0.003 (−0.136 to 0.142)	0.969	1.229
Education	0.578 (0.358 to 0.798)	<0.001	1.045
Hypertension	−0.068 (−2.906 to 2.770)	0.962	1.205
Diabetes	−0.644 (−2.967 to 1.679)	0.583	1.104
Smoking	0.078 (−2.746 to 2.902)	0.956	1.517
Alcohol abuse	1.526 (−2.102 to 5.154)	0.405	1.606
RCFT-C			
ALPS-index	5.153 (−10.967 to 21.274)	0.526	1.266
Age	−0.187 (−0.465 to 0.092)	0.186	1.181
Education	0.580 (0.140 to 1.019)	0.010	1.046
Hypertension	−2.081 (−7.841 to 3.678)	0.474	1.177
Diabetes	0.177 (−4.451 to 4.806)	0.939	1.090
Smoking	−0.255 (−5.892 to 5.382)	0.928	1.489
Alcohol abuse	−1.431 (−8.589 to 5.727)	0.692	1.556

*ALPS-index, diffusion tensor image analysis along the perivascular space; MoCA, Montreal Cognitive Assessment; AVLT-sum, total number of five times recall of three trial Auditory Verbal Learning Test; TMT (B-A), time of Trail Making Test B- time of Trail Making Test A; VFT, Category Verbal Fluency Test; SDMT, Symbol Digit Modalities Test; RCFT-C, Rey's Complex Figure Test copy trail; BNT, Boston Naming Test*.

Furthermore, after adjusting for the severity of WMLs, the number of lacunes, the number of CMBs and moderate to severe PVS, in the multivariate regression linear model, as shown in Table 4, the ALPS index was positively related to the MoCA (*p* = 0.048), AVLT-sum (*p* = 0.014), and SDMT (*p* = 0.001) and was negatively related to TMT (B-A; *p* = 0.041; Table 4). However, there were no significant linear associations among the VFT (*p* = 0.089), the BNT (*p* = 0.610), the RCFT-C (*p* = 0.663), and the ALPS index (Table 4).

**Table 4:** Multivariate regression linear analysis between neuropsychological test and imaging in CSVD.

	**B (95%CI)**	***P***	**VIF**
MoCA			
ALPS-index-index	8.423 (0.085 to 16.761)	0.048	1.144
WMLs	−1.577 (−2.332 to −0.823)	<0.001	1.307
Number of lacunes	−0.181 (−0.484 to 0.123)	0.241	1.173
Moderate to severe PVS	3.238 (−0.032 to 6.509)	0.052	1.026
Number of CMBs	−0.055 (−0.086 to −0.023)	0.001	1.098
TMT(B-A)			
ALPS-index	−179.413 (−351.329 to −7.496)	0.041	1.211
WMLs	11.508 (−1.942 to 24.959)	0.093	1.451
Number of lacunes	9.376 (2.666 to 16.085)	0.007	1.312
Moderate to severe PVS	31.974 (−32.038 to 95.986)	0.324	1.075
Number of CMBs	0.194 (−0.988 to 1.376)	0.746	1.293
AVLT-5sum		
ALPS-index	19.629 (3.967 to 35.292)	0.014	1.200
WMLs	−2.355 (−3.581 to −1.129)	<0.001	1.427
Number of lacunes	−0.169 (−0.779 to 0.441)	0.585	1.315
Moderate to severe PVS	−4.283 (−9.873 to 1.306)	0.132	1.049
Number of CMBs	−0.034 (−0.145 to 0.076)	0.539	1.279
SDMT			
ALPS-index	33.945 (15.206 to 52.684)	0.001	1.205
WMLs	−2.036 (−3.691 to −0.382)	0.016	1.307
Number of lacunes	−0.618 (−1.285 to 0.050)	0.069	1.231
Moderate to severe PVS	−1.187 (−8.439 to 6.065)	0.746	1.023
Number of CMBs	−0.043 (−0.162 to 0.076)	0.476	1.227
VFT			
ALPS-index	15.896 (−2.473 to 34.264)	0.089	1.125
WMLs	−2.449 (−4.088 to −0.811)	0.004	1.225
Number of lacunes	−1.026 (−1.740 to −0.312)	0.005	1.323
Moderate to severe PVS	7.163 (−0.068 to 14.394)	0.052	1.014
Number of CMBs	−0.118 (−0.256 to 0.020)	0.093	1.319
BNT			
ALPS-index	−1.722 (−8.388 to 4.944)	0.610	1.115
WMLs	−0.963 (−1.588 to −0.338)	0.003	1.262
Number of lacunes	−0.109 (−0.350 to 0.132)	0.372	1.153
Moderate to severe PVS	3.864 (1.185 to 6.542)	0.005	1.019
Number of CMBs	−0.042 (−0.067 to −0.016)	0.002	1.090
RCFT-C			
ALPS-index	2.859 (−10.115 to 15.833)	0.663	1.198
WMLs	−0.849 (−1.992 to 0.294)	0.144	1.277
Number of lacunes	−0.369 (−0.823 to 0.086)	0.111	1.210
Moderate to severe PVS	−1.467 (−6.718 to 3.783)	0.580	1.034
Number of CMBs	−0.060 (−0.139 to 0.019)	0.133	1.209

*ALPS-index, diffusion tensor image analysis along the perivascular space; WMLs, White Matter Lesions; CMBs, Cerebral Microbleeds; PVS, perivascular space; MoCA, Montreal Cognitive Assessment; AVLT-sum, total number of five times recall of three trial Auditory Verbal Learning Test; TMT (B-A), time of Trail Making Test B- time of Trail Making Test A; VFT, Category Verbal Fluency Test; SDMT, Symbol Digit Modalities Test; RCFT-C, Rey's Complex Figure Test copy trail; BNT, Boston Naming Test*.

## Discussion

Our results show that the ALPS index, which reflects the function of the glymphatic system, is related to cognitive impairment in patients with CSVD after adjusting for vascular risks and four classical CSVD markers, WMLs, lacunes, CMBs, and PVS.

Our results show that the decline in the ALPS index is independently related to cognitive impairment in patients with CSVD. In 2017, the author who proposed the ALPS index found that a decline in the ALPS index was correlated with a decrease in Mini-Mental State Examination (MMSE) scores in patients with AD (Taoka et al., 2017). This result was confirmed by another AD-related study that included only 36 participants (Steward et al., 2021). Using a battery of neuropsychological tests spanning multiple domains, a recent study also showed that the ALPS index in individuals in the Parkinson's disease (PD)-MCI and PD-dementia (PDD) groups was lower than that in the cognitively normal PD patient group (Chen et al., 2021). More recently, consistent with our results, a CSVD MRI study showed that the decline in the modified ALPS index in patients with CSVD and the modified ALPS index were independently related to MMSE scores in individuals in the CSVD cohort (Zhang et al., 2021).

The decline in the ALPS index with cognitive impairment is associated with the glymphatic system, which is reflected by this index and plays a vital role in the exchange between ISF and CSF in the brain. When ISF-CSF exchange is impaired, metabolic waste clearance, such as Aβ, in the brain is hindered, thus leading to neuronal damage and cognitive impairment (Wardlaw et al., 2020). In addition, the glymphatic system can carry immune cells (Mentis et al., 2021) and inflammatory factors (Carotenuto et al., 2021). As a result, dysfunction of the glymphatic system might result in neuroinflammation and neuronal damage (Carotenuto et al., 2021).

Our results show that both the ALPS index and WMLs are independent markers for CSVD cognitive impairment. The ALPS index calculated from DTI reflects the glymphatic system function, while WMLs are mainly a marker of demyelination. Thus, they represent different types of CSVD pathophysiology. However, the glymphatic system dysfunction is also associated with WMLs. First, the accumulation of toxic products and solutions caused by the glymphatic system dysfunction can damage the axon and lead to WMLs (Carotenuto et al., 2021). Second, arteriosclerosis and vascular stiffness in CSVD not only contribute to ischemia demyelination but also impair the impetus of the glymphatic system. In conclusion, the glymphatic system dysfunction and WMLs rooted in vascular damage have similar causes.

To the best of our knowledge, no study has analyzed the relationship between glymphatic system function and different cognitive domains in CSVD, and no study has assessed whether the ALPS index could be an independent marker to evaluate CSVD cognitive impairment. The present study used a multivariable logistic regression model to explore it. Aging and hypertension are confirmed CSVD risk factors, and the years of education are vital cognitive assessment scores. A recent study found a connection between diabetes and the ALPS index (Yang et al., 2020). Smoking leads to endothelial damage (Chang et al., 2014), and the latter triggers a series of cerebral small vessel dysfunctions (Wardlaw et al., 2013a). All those factors were added into the multivariable logistic regression model even though they did not reach statistical significance in univariate analysis. Our results show that the ALPS index, after vascular risk or traditional CSVD imaging marker adjustment, is linearly correlated with executive, attention, and memory function but is not correlated with language and visuospatial function.

The following principles may explain why the connection between ALPS index decrease and cognitive decline in CSVD is selective. First, vascular cognitive impairment selectively affects specific cognitive domains rather than homogeneously affecting all domains; numerous studies have demonstrated that executive function, attention, and memory are the main cognitive domains that are impaired in CSVD (Hamilton et al., 2021; Schroeter, 2021). Second, in murine studies in which a tracer was injected into the cerebellomedullary cistern, the tracer arrived in the dorsal and ventral brain asynchronously (Iliff et al., 2013a; Zeppenfeld et al., 2017; Mortensen et al., 2019). This phenomenon suggests that the speed of metabolic clearance based on the glymphatic system varies in different areas. The difference ultimately influences cerebral metabolism. Third, the glymphatic system derives motives from arterial pulsation (Iliff et al., 2013b; Mestre et al., 2018). Differences in arterial remodeling and hemodynamics of superficial and deep arterioles may influence glymphatic circulation in corresponding brain areas (Blanco et al., 2017; Spence, 2019). This difference finally has an effect on cerebral metabolism. Last, the ALPS index data are based only on measurements taken beside the body of the lateral ventricle, which includes a variety of connections to different cortical areas. This may affect the validity of cognitive domain evaluations.

Our study had some limitations. First, even though the emerging ALPS index has been confirmed by a study comparing the ALPS index method to the gold standard method, the ALPS index method is not a direct way to evaluate the glymphatic system and needs to be verified by more studies in the future. Second, this study was a single-center study, and the sample size was relatively small. This might be the main reason why the severity of PVS or the number of lacunes in patients in the CSVD-CN group was not significantly different from that in patients in the CSVD-CI group. Third, considering the relatively small sample size, we did not adjust for all vascular risk factors in the multivariate analysis. This may influence the reliability of our research.

## Conclusion

In conclusion, our study has shown that a decrease in the ALPS index is independently associated with executive, attention, and memory function impairment in patients with CSVD, and further studies should explore whether the ALPS index could predict cognitive decline in these patients.

## Data Availability Statement

The datasets presented in this article are not readily available because, we do not have permission to share them. Requests to access the datasets should be directed to JF, jianhuifu@126.com.

## Ethics Statement

This study was approved by the Institutional Review Board of Huashan Hospital. The patients/participants provided their written informed consent to participate in this study.

## Author Contributions

JT: methodology, formal analysis, investigation, project administration, data curation, writing—original draft, writing—review and editing, and funding acquisition. MZ: resources, investigation, project administration, data curation, and writing—review and editing. NL, YX, XR, and QH: investigation and writing—review and editing. LS: resources, project administration, and writing—review and editing. JF: conceptualization, methodology, writing—review and editing, supervision, and funding acquisition. All authors contributed to the article and approved the submitted version.

## Funding

Funding support was provided by the National Natural Science Foundation of China (81901179, JT) and the Scientific Research Plan Project of Shanghai Science and Technology Committee (18411962100, JF). JT was supported by the Huaxiu Talents Program of Huashan Hospital (2020).

## Conflict of Interest

The authors declare that the research was conducted in the absence of any commercial or financial relationships that could be construed as a potential conflict of interest.

## Publisher's Note

All claims expressed in this article are solely those of the authors and do not necessarily represent those of their affiliated organizations, or those of the publisher, the editors and the reviewers. Any product that may be evaluated in this article, or claim that may be made by its manufacturer, is not guaranteed or endorsed by the publisher.
